# Suicin 3908, a New Lantibiotic Produced by a Strain of *Streptococcus suis* Serotype 2 Isolated from a Healthy Carrier Pig

**DOI:** 10.1371/journal.pone.0117245

**Published:** 2015-02-06

**Authors:** Katy Vaillancourt, Geneviève LeBel, Michel Frenette, Marcelo Gottschalk, Daniel Grenier

**Affiliations:** 1 Groupe de Recherche en Écologie Buccale (GREB), Faculté de Médecine Dentaire, Université Laval, Quebec City, QC, Canada; 2 Centre de Recherche en Infectiologie Porcine et Avicole (CRIPA), Fonds de Recherche du Québec—Nature et Technologies (FRQNT), Quebec City, QC, Canada; 3 Groupe de Recherche sur les Maladies Infectieuses du Porc (GREMIP), Faculté de Médecine Vétérinaire, Université de Montréal, Saint-Hyacinthe, QC, Canada; Centers for Disease Control & Prevention, UNITED STATES

## Abstract

While *Streptococcus suis* serotype 2 is known to cause severe infections in pigs, it can also be isolated from the tonsils of healthy animals that do not develop infections. We hypothesized that *S. suis* strains in healthy carrier pigs may have the ability to produce bacteriocins, which may contribute to preventing infections by pathogenic *S. suis* strains. Two of ten *S. suis* serotype 2 strains isolated from healthy carrier pigs exhibited antibacterial activity against pathogenic *S. suis* isolates. The bacteriocin produced by *S. suis* 3908 was purified to homogeneity using a three-step procedure: ammonium sulfate precipitation, cationic exchange HPLC, and reversed-phase HPLC. The bacteriocin, called suicin 3908, had a low molecular mass; was resistant to heat, pH, and protease treatments; and possessed membrane permeabilization activity. Additive effects were obtained when suicin 3908 was used in combination with penicillin G or amoxicillin. The amino acid sequence of suicin 3908 suggested that it is lantibiotic-related and made it possible to identify a bacteriocin locus in the genome of *S. suis* D12. The putative gene cluster involved in suicin production by *S. suis* 3908 was amplified by PCR, and the sequence analysis revealed the presence of nine open reading frames (ORFs), including the structural gene and those required for the modification of amino acids, export, regulation, and immunity. Suicin 3908, which is encoded by the *suiA* gene, exhibited approximately 50% identity with bovicin HJ50 (*Streptococcus bovis*), thermophilin 1277 (*Streptococcus thermophilus*), and macedovicin (*Streptococcus macedonicus*). Given that *S. suis* 3908 cannot cause infections in animal models, that it is susceptible to conventional antibiotics, and that it produces a bacteriocin with antibacterial activity against all pathogenic *S. suis* strains tested, it could potentially be used to prevent infections and to reduce antibiotic use by the swine industry.

## Introduction

*Streptococcus suis* is a major swine pathogen that has been associated with severe infections such as meningitis, arthritis, endocarditis, and septicemia [1]. It is one of the most important bacterial pathogens responsible for major economic losses in the swine industry worldwide. In addition, this Gram-positive bacterium is recognized as an emerging zoonotic agent for humans exposed to sick pigs or their by-products and has caused major outbreaks in Asia [2]. Of the 35 recognized serotypes of *S*. *suis*, serotype 2 is the serotype most frequently isolated from diseased pigs [1].

Antibiotics have been extensively used in the swine industry as growth promoters, as well as for prophylactic and metaprophylactic treatments to prevent and treat infections [3, 4]. This use has likely contributed to the spread of antibiotic resistance, more particularly resistance to macrolides, lincosamides, sulphonamides, and tetracyclines, in *S*. *suis* [4, 5]. Resistant strains could then spread from animals to humans either directly or indirectly through the food chain or through more complex environmental routes of exposure. Bacteria can also transfer resistance genes to other bacterial species in various ecosystems. Since this can compromise the treatment of human infections, alternative therapeutic and preventive strategies for *S*. *suis* infections need to be identified. In this regard, bacteriocins, which are ribosomally synthesized antimicrobial peptides of bacterial origin, are of great interest [6]. Bacteriocins may be either active on a number of different bacterial species or target specific species by inducing the formation of membrane pores or acting on other essential cellular components [7]. A protective immunity system ensures that the bacteriocin-producing strains are resistant to their own bacteriocin [8]. The lantibiotic nisin A, which is currently used commercially as a food preservative, especially in dairy products in more than 50 countries [9], has been reported to be active against *S*. *suis* and to potentiate the activity of conventional antibiotics used in the swine industry [10]. We recently reported that a non-virulent strain of *S*. *suis* (serotype 2) secreted a bacteriocin (suicin 90–1330) that is active against pathogenic *S*. *suis* isolates [11]. Suicin 90–1330, is a lantibiotic that exhibits high homology with nisin U [11].

Although *S*. *suis* serotype 2 can cause severe infections in pigs, it can also be isolated from the tonsils of healthy animals that do not develop infections [12–14]. Zhang et al. [13] reported that 40.4% and 3.0% of clinically healthy animals in swine herds in China harbor *S*. *suis* and serotype 2 in their tonsils, respectively. It has been estimated that 43.6% of slaughter pigs in Canada are carriers (tonsils) of *S*. *suis*, while 7.0% harbor serotype 2 [14]. In the present study, we hypothesized that *S*. *suis* serotype 2 strains recovered from healthy carrier pigs may have the ability to produce bacteriocins and may thus be potentially interesting from a therapeutic/preventive point of view. The aim of our study was to purify and characterize a bacteriocin produced by a strain of *S*. *suis* serotype 2 isolated from a healthy carrier pig.

## Materials and Methods

### Bacterial strains and growth conditions

All *S*. *suis* serotype 2 strains used in this study as well as their origins are listed in Table 1. These strains were obtained from the bacterial culture collection of Dr. M. Gottschalk (Université de Montréal, St-Hyacinthe, QC, Canada). They were routinely grown under static conditions at 37°C in Todd Hewitt broth (THB; BBL Microbiology Systems, USA).

**Table 1:** *S*. *suis* serotype 2 strains used in this study.

Strain	Country	Origin
3908	The Netherlands	Healthy carrier pig^1^
3978	The Netherlands	Healthy carrier pig
4/3H1	Canada	Healthy carrier pig
4/39H1	Canada	Healthy carrier pig
4/40H2	Canada	Healthy carrier pig
94–623	France	Healthy carrier pig
B268α	Canada	Healthy carrier pig
DAT292	Japan	Healthy carrier pig
T15	The Netherlands	Healthy carrier pig
TD10	United Kingdom	Healthy carrier pig
31533	France	Meningitis (ST1)
DAT229	Japan	Endocarditis (ST1)
MGGUS3	United States	Meningitis (ST1)
MNCM06	Thailand	Meningitis (ST1)
P1/7	United Kingdom	Meningitis (ST1)
P517/03P4	Argentina	Meningitis (ST1)
1043248	Canada	Meningitis (ST25)
MGGUS4	United States	Septicemia (ST25)
1054471	Canada	Meningitis (ST28)
MGGUS10	United States	Pneumonia (ST28)

^1^ All strains from healthy carrier pigs were isolated from tonsils.

### Plate diffusion assay for detecting bacteriocin production

Overnight cultures of *S*. *suis* from ten healthy carrier pigs were spotted (2 µL) on THB agar plates. After a 24-h incubation at 37°C, the plates were overlaid with soft THB agar (0.75%, w/v) that had been inoculated (700 µL of culture/7 mL of agar) with a 24-h culture of a pathogenic indicator strain of *S*. *suis*, and were further incubated for 24 h at 37°C. The inhibition zones (in mm) were measured from the edge of the *S*. *suis* growth to the margin of the inhibitory zones.

### Detection of the *sslA* gene

The bacteriocin-producing strains were tested by PCR for the presence of the *sslA* structural gene previously identified in the bacteriocin-producing strain *S*. *suis* 90–1330 [11]. PCR reactions consisted of 40.8 *μ*L of PCR grade water, 5 *μ*L of 10× reaction buffer, 1 *μ*L of nucleotide mix, 0.6 *μ*L each of the appropriate forward and reverse primers for *sslA* (G48: 5’-AAACAACTCAGGAGCTTCAC-3’ and G130R: 5’-CACAGGTCATCAAAATACCC-3’, respectively) or *gdh* (used as a positive control) (GDH645: 5’-TTTGGTTTACTTCACTGATAACATG-3’ and GDH794R: 5’-GAGTCTGAAACAGAAATAACTTTTG-3’, respectively), 1 *μ*L of EconoTaq DNA polymerase (5 U/*μ*L), and 1 *μ*L of genomic DNA as a template. The PCR was performed with a DNA Thermal Cycler 480 (Perkin-Elmer, USA) according to the EconoTaq reaction protocol of Lucigen Corporation (USA). The reaction was carried out for 20 cycles with the following temperature-time profile: 95°C for 1 min, 55°C for 1 min, and 72°C for 30 s. At the end of the amplification protocol, the samples were incubated at 72°C for 3 min. A 1% agarose gel was used to analyze the PCR products.

### Purification of bacteriocin produced by *S*. *suis* 3908

*S*. *suis* 3908 was cultivated in 2 L of a culture medium containing 2% proteose-peptone, 1% yeast extract, 0.25% glucose, 0.25% NaCl, 0.3% K_2_HPO_4_, 0.2% KH_2_PO_4,_ 0.01% MgSO_4_ · 7 H_2_O, and 0.002% MnSO_4_ · 7 H_2_O (pH 7.0). This medium, which increases bacteriocin production by *S*. *suis* [11], was supplemented with 0.01% Tween 80 (sorbitan polyoxyethylene monooleate; Sigma-Aldrich, Canada) to prevent bacteriocin adsorption to glassware and bacterial cells and to minimize bacteriocin loss [15]. Following incubation for 24 h at 37°C under aerobic conditions, bacterial cells were removed by centrifugation (10,000 x *g* for 15 min at 4°C). Ammonium sulfate was slowly added to the culture supernatant to obtain 50% saturation, and the mixture was stirred for 3 h at 4°C. The supernatant was centrifuged (14,000 x *g* for 10 min at 4°C), and the precipitate was suspended in 30 mL of 50 mM phosphate-buffered saline pH 7.2 (PBS) and dialyzed (1000 Da cut-off) overnight against 20 mM 2-(*N*-morpholino)ethanesulfonic acid (MES) buffer (pH 5.5) containing 0.01% Tween 80. The dialysate was eluted on a MonoS 5/50 GL cationic exchange high-pressure liquid chromatography column (GE Healthcare, Canada) using an ÄKTA Purifier system (GE Healthcare). The elution was performed at a flow rate of 1 mL/min using a linear 0 to 0.5 M gradient of KCl. The active fractions were detected using the spot test assay, which consists in spotting 5 µL on the surface of THB agar plates inoculated with a lawn of the *S*. *suis* MGGUS3 (sequence type1 (ST1), as determined by multilocus sequence typing). The plates were incubated for 24 h at 37°C, and the fractions with an inhibitory zone were pooled and were dialyzed (1000 Da cut-off) overnight against 0.01% trifluoroacetic acid (TFA) + 10% acetonitrile. The pooled fraction was then eluted on a SOURCE 15RPC column (GE Healthcare) at a flow rate of 1 mL/min using a linear gradient of 10 to 90% acetonitrile. The acetonitrile and TFA were removed by a rotary evaporator prior to analyzing the fractions for bacteriocin activity using the spot test plate assay. The active fractions were pooled, and glycerol was added to a final concentration of 15%. The pooled fraction was then aliquoted (100 µL) and stored at—80°C until used. Antibacterial activity of the final purified bacteriocin fraction was quantified in arbitrary units, which correspond to the reciprocal of the highest two-fold serial dilution giving a clear inhibitory zone following the deposition of 5 µL of the bacteriocin solution on a lawn of *S*. *suis* MGGUS3. Total purified bacteriocin was also quantified using Quick Start Bradford protein assay kits (Bio-Rad Laboratories, Canada).

### SDS-PAGE analysis

The purified bacteriocin preparation (0.3 µg) was separated on 4–20% TGX™ gels (Bio-Rad), and the gels were fixed in 10% acetic acid/40% methanol (30 min) prior to be stained with Coomassie blue. One gel was also fixed in 10% acetic acid:20% propanol (30 min) and was washed thoroughly in sterile distilled water (3 x 30 min). Bacteriocin activity was detected using an overlay of soft agar medium inoculated with the indicator strain *S*. *suis* MGGUS3. Nisin A (Sigma-Aldrich) was used as a positive control.

### Bacteriocin characterization

The susceptibility of the purified bacteriocin to heat, pH, and enzymatic treatments was determined using the spot test plate assay and *S*. *suis* MGGUS3 as the indicator strain. To evaluate its temperature stability, the purified bacteriocin was incubated at 45, 70, 100, or 121°C for 15 min and at 4°C or room temperature for one week. To evaluate its susceptibility to extreme pHs, the bacteriocin solution was adjusted to pH 2 or 11 with 0.125 N HCl or 0.125 N NaOH, respectively, and was incubated for 15 min at room temperature. Lastly, the susceptibility of the bacteriocin to proteolytic cleavage was evaluated using trypsin, chymotrypsin, and proteinase K (Sigma-Aldrich), each at a final concentration of 500 µg/mL. The solutions were incubated for 1 h at 37°C and were treated for 5 min at 70°C to inactivate the proteolytic enzymes.

### Inhibitory spectrum of the purified bacteriocin

Several swine pathogens were tested using the spot test plate assay to evaluate the inhibitory spectrum of the purified bacteriocin. *Actinobacillus pleuropneumoniae* 81–750 and *Pasteurella multocida* ATCC 12948 were grown on THB agar plates supplemented with nicotinamide adenine dinucleotide (NAD; 20 µg/mL), *Actinobacillus suis* JG-2 was grown on THB blood agar plates, *Haemophilus parasuis* 99–9048-B and *Bordetella bronchiseptica* ATCC 19395 were grown on THB agar plates supplemented with NAD (20 µg/mL) and hemin (10 µg/mL) or NAD (20 µg/mL) and cysteine (400 µg/mL), and *Escherichia coli* P82–862, *Staphylococcus aureus* ATCC 25923, and *Staphylococcus hyicus* ATCC 11249 were grown on THB agar plates. The plates were incubated for 24 h at 37°C in aerobic conditions.

### Fractional inhibitory concentration index of the purified bacteriocin in combination with antibiotics

The effect of the purified bacteriocin in combination with penicillin G or amoxicillin was evaluated using the checkerboard technique [16]. The purified bacteriocin was serially diluted in THB (100 µL) along the ordinate of a 96-well microplate, while the antibiotics were serially diluted in THB (100 µL) along the abscissa. A cell suspension of *S*. *suis* MGGUS3 (ST1) prepared in THB and adjusted to an OD_660_ of 0.2 was used as inoculum. The wells were inoculated with 100 µL of the bacterial suspension, and the microplate was incubated for 24 h at 37°C. Wells with no bacteria or compounds were used as controls. Bacterial growth was assessed visually. The lowest concentration at which no growth occurred was considered the MIC. The fractional inhibitory concentration index (FICI) was calculated using the following equation:$\text{FICI}={\text{FIC}}_{\text{A}}+{\text{FIC}}_{\text{B}}=\frac{\left({\text{MIC}}_{\text{Antibiotic}}\text{in combination}\right)}{\left({\text{MIC}}_{\text{Antibiotic}}\text{alone}\right)}+\frac{\left({\text{MIC}}_{\text{Bacteriocin}}\text{in combination}\right)}{\left({\text{MIC}}_{\text{Bacteriocin}}\text{ alone}\right)}$. An FICI ≤ 0.5 was considered as indicating a synergistic effect, an FICI > 0.5 and ≤ 1.0 as indicating an additive effect, an FICI > 1.0 and ≤ 4.0 as indicating no effect, and an FICI > 4.0 as indicating an antagonistic effect. Two independent experiments were performed to ensure reproducibility.

### Membrane permeabilization assay

The ability of the purified bacteriocin to permeabilize the cytoplasmic membrane of *S*. *suis* MGGUS3 was evaluated as previously described [11] using SYTOX Green dye (Life Technologies Inc., Canada), which binds to the nucleic acid of bacterial cells once the cytoplasmic membrane has been compromised. The fluorescence resulting from the binding of the dye to the bacterial DNA was recorded using a Synergy 2 microplate reader (BioTek Instruments, USA) every 3 min for 20 min with the excitation and emission wavelengths set at 485 nm and 528 nm, respectively. Ethanol (70%) was used as a positive control. PBS was used as a negative control. Three assays were performed, and the means ± standard deviations were calculated.

### Amino acid sequencing

The purified bacteriocin was subjected to SDS-PAGE as described above and was then electroblotted onto a polyvinylidene difluoride (PVDF) membrane. The bacteriocin band, which was located based on the migration of molecular weight markers and the detection of bacteriocin activity, was excised and transferred into a microtube. Ethanethiol derivatization of post-translationally modified amino acids of the PVDF-blotted bacteriocin was carried out as previously described by Meyer et al. [17]. A reaction mixture (200 μL) composed of 280 μL of methanol, 200 μL of H_2_O, 65 μL of 5 M NaOH, and 60 μL of ethanethiol was added to the microtube containing the bacteriocin band. Following a 1-h incubation at 50°C in an oxygen-free atmosphere, the reaction mixture was acidified by adding 66 μL of 70% (v/v) formic acid, and the bacteriocin band was vacuum-dried. The band was then sent to the SPARC BioCentre (The Hospital for Sick Children, Toronto, ON, Canada), and the bacteriocin was subjected to Edman degradation using an Applied Biosystems ABI 492 Procise cLC sequencer (Life Technologies Inc.).

### Identification of the putative gene cluster encoding bacteriocin 3908

Published *S*. *suis* genomes were blasted for the presence of proteins related to the bacteriocin amino acid sequence using the UniPROT web-based platform (http://www.uniprot.org) [18]. The putative bacteriocin gene cluster that was identified was used to design primers for PCR amplification using genomic DNA extracted from *S*. *suis* 3908. The sequences of the forward and reverse primers were A284: 5’- CAAACTGCAACTGATCAAGAAATTA-3’ and A398R: 5’-AATTTTTGCACCCAGAGATGAATCC-3’, respectively. The putative bacteriocin 3908 locus as well as the BLAST homolog were then sequenced.

### Immunity assay

Bacterial strains that produce either identical or similar bacteriocins exhibit cross-immunity. Therefore, the presence of cross-immunity between bacteriocin producers is generally indicative of relatedness between their bacteriocins. To evaluate cross-immunity, *S*. *suis* 90–1330 and 3908, which produce suicin 90–1330 and suicin 3908, respectively, were used as either bacteriocin producers or indicators in the plate diffusion assay.

### Antibiotic susceptibility of *S*. *suis* 3908

The susceptibility of *S*. *suis* 3908 to antibiotics (amoxicillin, ampicillin, ceftiofur, erythromycin and penicillin G) was determined as follows. Briefly, a 24-h bacterial culture in THB was diluted in fresh broth medium to obtain an optical density at 660 nm (OD_660_) of 0.2. Equal volumes (100 µL) of bacteria and serial dilutions of antibiotics in THB were mixed in the wells of 96-well plates. Wells with no bacteria or no antibiotics were used as controls. Following a 24-h incubation at 37°C, bacterial growth was recorded visually. Minimal inhibitory concentration (MIC) values (μg/mL) were expressed as the lowest concentration at which no growth occurred. The MIC values were determined in three independent experiments.

## Results

**Fig 1:**
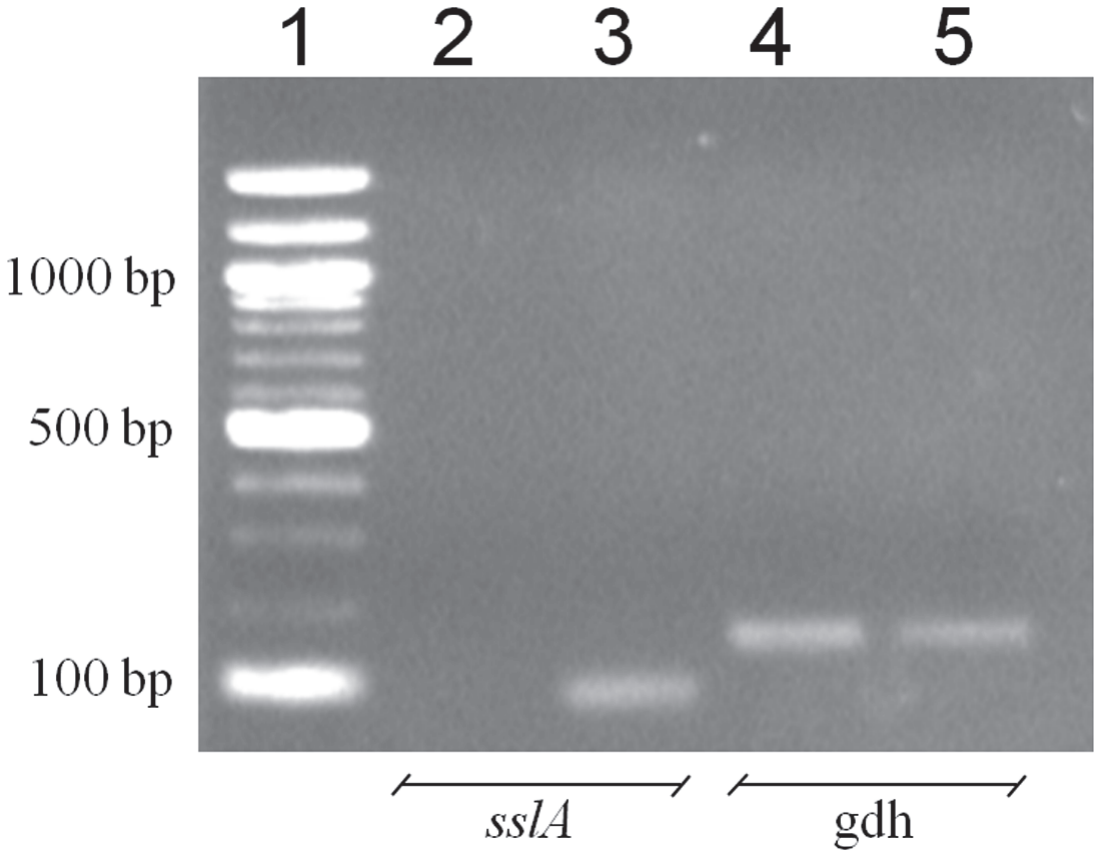
Detection of *sslA*, the structural gene of suicin 90–1330, in *S*. *suis* 3908 and 94–623, two bacteriocin-producing strains. Lane 1, DNA molecular weight markers; lanes 2 and 4, *S*. *suis* 3908; lanes 3 and 5, *S*. *suis* 94–623; lanes 2 and 3, *sslA* gene; lanes 4 and 5, *gdh* gene.

Two of the ten strains of *S*. *suis* (3908 and 94–623) isolated from healthy carrier pigs exhibited strong antibacterial activity (5-mm inhibitory zone) against *S*. *suis* MGGUS3, a pathogenic isolate of ST1 used as the indicator strain. Given that we previously purified and characterized a lantibiotic (suicin 90–1330) produced by a strain of *S*. *suis*, we used PCR to detect the presence of the structural gene (*sslA*) coding for suicin 90–1330 in the two new producing strains. As shown in Fig. 1, strain 94–623 possessed the *sslA* gene while the gene was not amplified in strain 3908. The *gdh* gene used as control was amplified in both strains. This confirmed that the bacteriocin produced by *S*. *suis* 3908 was not the suicin 90–1330. We thus selected this strain for further analysis.

**Fig 2:**
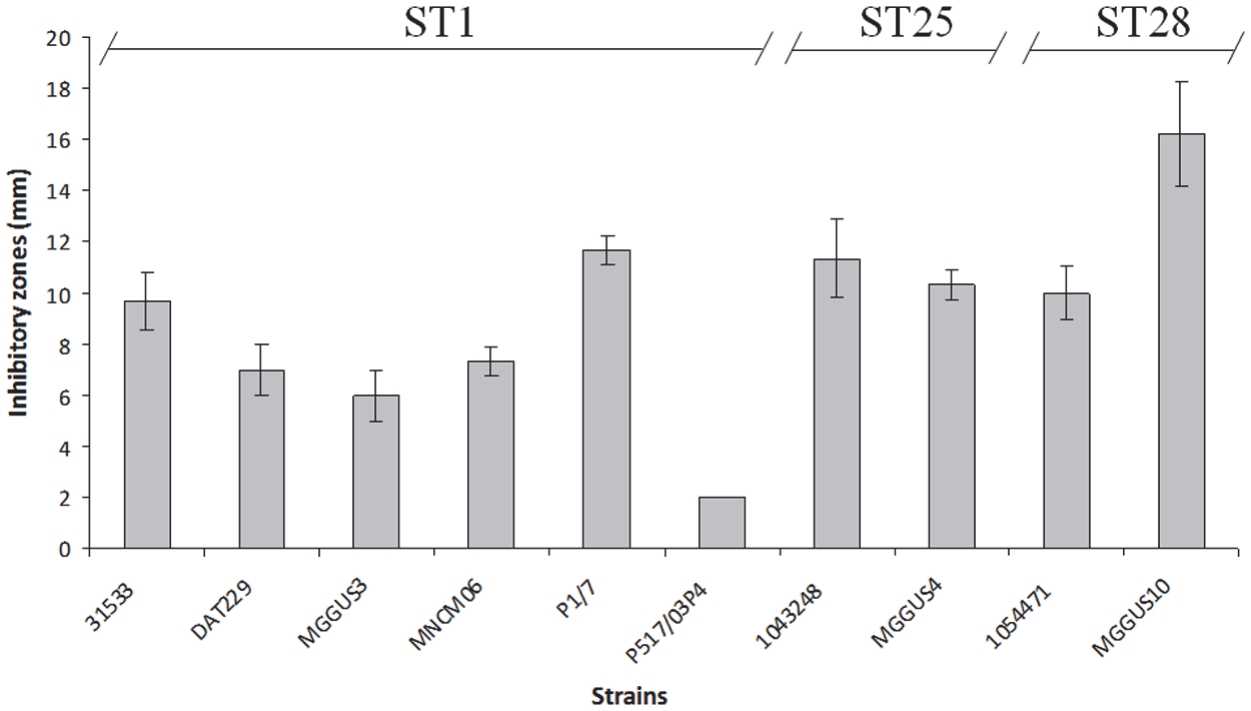
Inhibitory zones produced by *S*. *suis* 3908 when spotted on lawns of *S*. *suis* strains belonging to different sequence types (ST1, ST25, ST28) and that were isolated from diseased pigs. Three independent experiments were performed. The inhibitory zones (in mm) are expressed as means ± standard deviations.

We used a plate diffusion assay to test *S*. *suis* 3908 for its capacity to inhibit a large array of *S*. *suis* strains isolated from diseased pigs. These strains belonged to either ST1, ST25, or ST28, which are known to be highly, moderately, and slightly virulent in animal models, respectively [19] (unpublished data). As shown in Fig. 2, *S*. *suis* 3908 produced inhibitory zones of various sizes (2 to 16 mm) against all the strains tested (n = 10). No relationship was observed between specific STs and higher or lower susceptibility.

**Fig 3:**
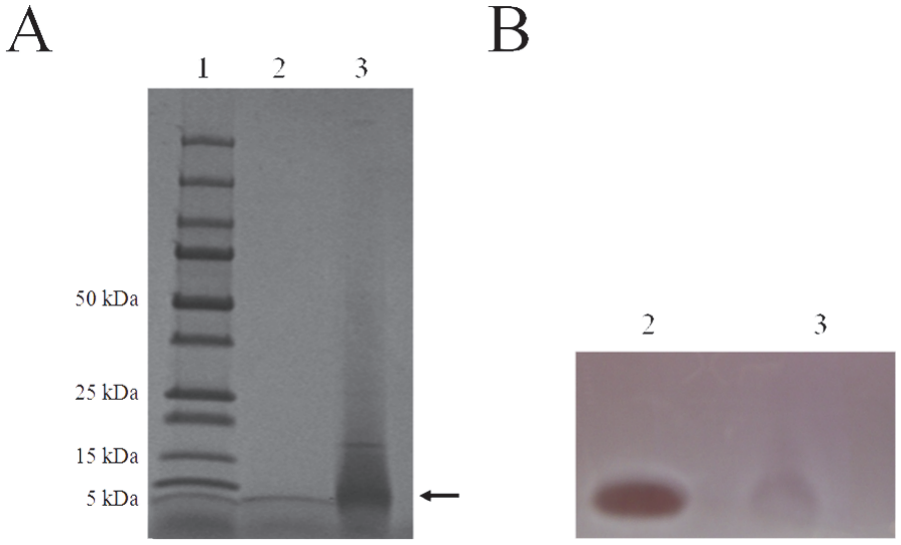
SDS-PAGE analysis of the purified bacteriocin produced by *S*. *suis* 3908. Panel A. Gel stained with Coomassie blue. Panel B. Gel used to detect antibacterial activity by an overlay with the indicator strain *S*. *suis* MGGUS3. Lane 1: molecular weight markers; Lane 2: nisin A; Lane 3: suicin 3908.

The bacteriocin produced by *S*. *suis* 3908 was purified from a 2-L culture supernatant using a simple three-step procedure: ammonium sulfate precipitation, cationic exchange HPLC, and reversed-phase HPLC. Bacteriocin activity recovered from the reversed-phase HPLC column eluted in a single peak. The peak yielded a single Coomassie blue-stained band on an SDS-PAGE gel and migrated the same distance as commercial lantibiotic nisin A, which has a molecular mass of 3,354 Da (Fig. 3A). An overlay of the polyacrylamide gel with the indicator strain (MGGUS3) correlated bacteriocin activity with the protein band (Fig. 3B). The purification protocol recovered 891 µg of protein from the 2-L *S*. *suis* 3908 culture with 64,800 arbitrary units (AU) of bacteriocin, which corresponded to a specific activity of 72.7 AU/µg of protein.

The purified suicin 3908 was subjected to various treatments, and its activity was evaluated using the spot test plate assay to determine its stability (Table 2). Antibacterial activity was detected even after a 15-min treatment at 121°C, indicating that the bacteriocin is highly heat stable. In addition, storage at room temperature for one week did not reduce the antibacterial activity. Extreme pHs (2 and 11) had no effect on the stability of suicin 3908. Lastly, proteolytic enzymes (trypsin, chymotrypsin, proteinase K) had no detectable effect on antibacterial activity. To determine its inhibitory spectrum, suicin 3908 was tested against various Gram-positive and Gram-negative bacterial species known to cause infections in swine. As shown in Table 2, suicin 3908 was only active against *S*. *hyicus*.

**Table 2:** Stability and activity spectrum of suicin 3908.

Treatment	Indicator strain	Inhibitory activity
45°C / 15 min	*S*. *suis* MGGUS3	+
70°C / 15 min	*S*. *suis* MGGUS3	+
100°C / 15 min	*S*. *suis* MGGUS3	+
121°C / 15 min	*S*. *suis* MGGUS3	+
4°C / 7 d	*S*. *suis* MGGUS3	+
25°C / 7 d	*S*. *suis* MGGUS3	+
pH 2 / 15 min	*S*. *suis* MGGUS3	+
pH 11 / 15 min	*S*. *suis* MGGUS3	+
Trypsin / 60 min	*S*. *suis* MGGUS3	+
Chymotrypsin / 60 min	*S*. *suis* MGGUS3	+
Proteinase K / 60 min	*S*. *suis* MGGUS3	+
None	*S*. *aureus* ATCC 25923	-
None	*S*. *hyicus* ATCC 11249	+
None	*A*. *pleuropneumoniae* 81–750	-
None	*A*. *suis* JG-2	-
None	*B*. *bronchiseptica* ATCC 19395	-
None	*E*. *coli* P82–862	-
None	*H*. *parasuis* 99–9048-B	-
None	*P*. *multocida* ATCC 12948	-

The MIC of suicin 3908 against *S*. *suis* MGGUS3 was determined and was compared to that of commercial nisin A to assess its relative antibacterial activity. Suicin 3908 had a MIC of 0.47 µg/mL while that of nisin A was 0.156 µg/mL. We used the checkerboard technique to determine whether suicin 3908 in combination with antibiotics had a synergistic or additive effect against *S*. *suis* MGGUS3. Suicin 3908 in association with penicillin G or amoxicillin gave a FICI of 1, which indicates an additive effect.

**Fig 4:**
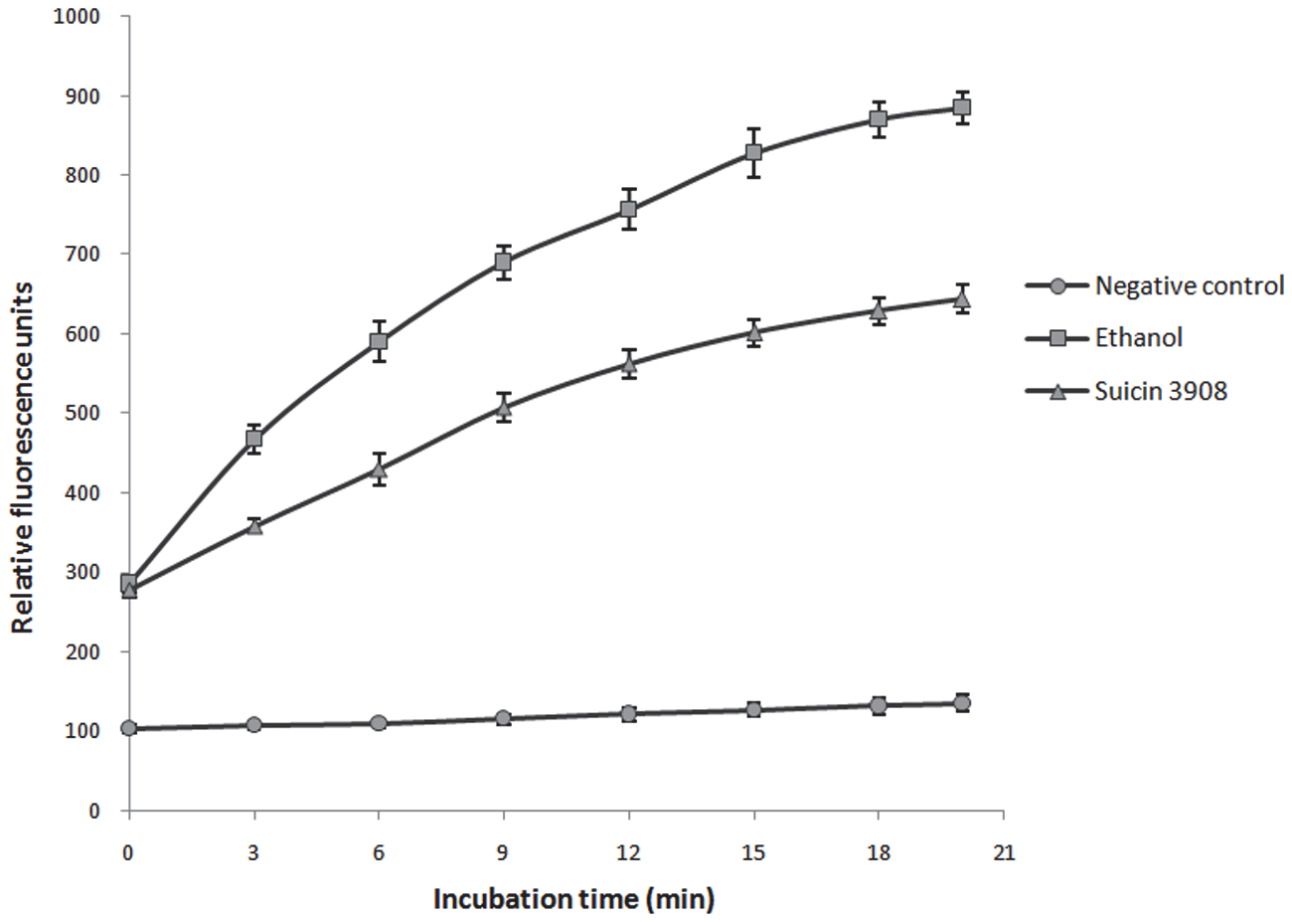
Effect of suicin 3908 on the membrane permeabilization of *S*. *suis* MGGUS3 as determined using the SYTOX Green dye. Ethanol (70%) was used as a positive control. Three assays were performed. The results are expressed as means ± standard deviations.

The ability of suicin 3908 to permeabilize the membrane of the indicator strain *S*. *suis* MGGUS3 was investigated using SYTOX Green dye, which can penetrate damaged cytoplasmic membranes and react with DNA. As shown in Fig. 4, the addition of either the purified bacteriocin or ethanol (positive control) resulted in a time-dependent increase in fluorescence, indicating that the membrane of *S*. *suis* MGGUS3 was permeabilized by suicin 3908. No increase in fluorescence occurred with the negative control.

**Fig 5:**
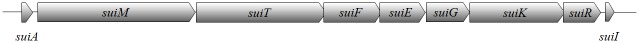
Genetic organization of the putative suicin 3908 gene cluster.

The purified bacteriocin was derivatized with ethanethiol and was then subjected to Edman degradation. An analysis of the first ten amino acids of the purified bacteriocin yielded the following sequence: Ala_1_-Gly_2_-Ser_3_-Gly_4_-Phe_5_-Val_6_-Lys_7_-X_8_-Leu_9_-X_10_-, where the X residues are modified cysteines, serines, or threonines. Based on a blast search using the UniPROT web-based platform (http://www.uniprot.org) [18], the ten-amino acid sequence shared perfect inentity with the N-terminus of a lantibiotic (accession no. G7SEC9) produced by *S*. *suis* D12. An analysis of the *S*. *suis* D12 genome revealed the presence of a complete lantibiotic biosynthesis locus. Based on the gene locus in *S*. *suis* D12, primers were designed for PCR amplification of *S*. *suis* 3908. Fig. 5 shows the complete *suiA* gene cluster identified in *S*. *suis* 3908. The cluster contained nine ORFs involved in bacteriocin production. More specifically, the cluster contained ORFs for the suicin 3908 precursor (*suiA*), a synthetase involved in lantibiotic modification (*suiM*), an ABC transporter (*suiT*), a response regulator (*suiR*), a sensor histidine kinase (*suiK*), and four immunity proteins (*suiF*, *suiE*, *suiG*, and *suiI*).

**Fig 6:**
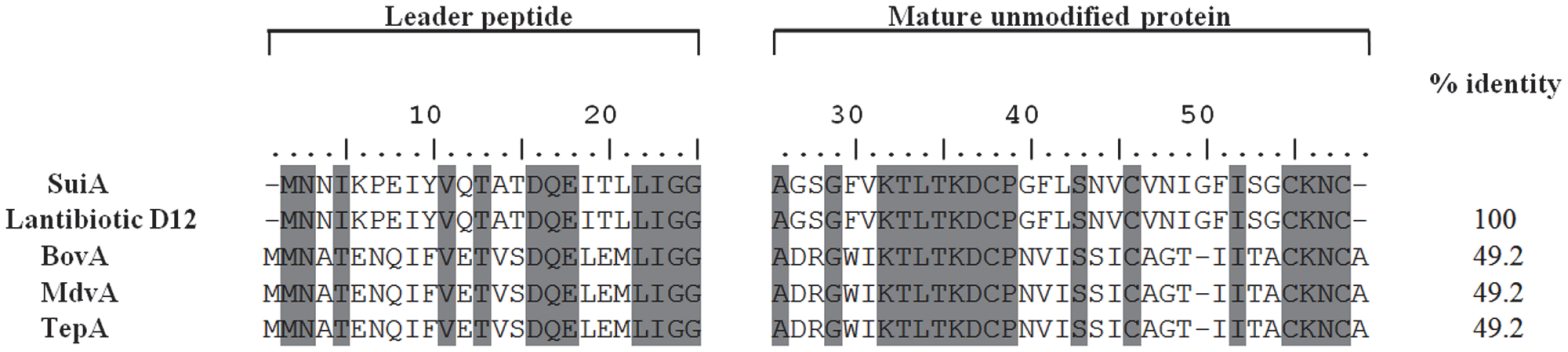
Comparison of the amino acid sequence of suicin 3908 with the amino acid sequence of the putative lantibiotic corresponding to gene SSUD12_1310 (accession # G7SEC9) in *S*. *suis* D12 and with other lantibiotics produced by *S*. *bovis* (bovicin HJ50; *bovA*), *S*. *macedonicus* (macedovicin; *mdvA*), and *S*. *thermophilus* (thermophilin 1177; *tepA*).

Based on the structural gene (*suiA*) of suicin 3908, the inferred amino acid sequence of the leader peptide contained 24 amino acids, while that of mature unmodified peptide consisted of 33 amino acids (Fig. 6). A comparison of the SuiA amino acid sequence with previously characterized lantibiotics revealed that it shares approximately 50% identity with bovicin HJ50, thermophilin 1277, and macedovicin, which are produced by *Streptococcus bovis*, *Streptococcus thermophilus*, and *Streptococcus macedonicus*, respectively (Fig. 6). Immunity peptides are known to confer specific protection against the cognate bacteriocin [8]. As expected, since the bacteriocin produced by *S*. *suis* 90–1330 and *S*. *suis* 3908 exhibited poor identity (22%), no cross-immunity was observed between the two bacteriocin-producing strains (data not shown).

Given that the bacteriocin-producing *S*. *suis* 3908 may have potential probiotic and protective applications, we were interested in evaluating its susceptibility to antibiotics. *S*. *suis* 3908 was highly sensitive to amoxicillin and erythromycin with a MIC of 0.00975 µg/mL as well as to penicillin G and ceftiofur with a MIC of 0.0195 µg/mL. The strain was less sensitive to ampicillin with a MIC of 0.156 µg/mL.

## Discussion

Antibiotics are commonly used in the swine industry to prevent disease and promote growth [3, 4]. Consequently, *S*. *suis* strains that are resistant to a number of antibiotics, including macrolides, tetracyclines, and lincosamides, are now frequently isolated [4, 5]. Since *S*. *suis* could serve as a reservoir and transfer resistance genes to human pathogens, the identification of alternative therapeutic and preventive strategies for infections caused by *S*. *suis* has become a priority. One strategy to overcome this problem is to search for new antimicrobial agents. In this regard, the use of bacteriocins, which are ribosomally synthesized antimicrobial peptides of bacterial origin with a narrow or broad activity spectrum, may represent a promising and effective strategy for the prevention/treatment of *S*. *suis* infections [6].

LeBel et al. [11] recently purified a bacteriocin (suicin 90–1330) produced by *S*. *suis* 90–1330, an ST28 strain that is non-virulent in mouse and pig models of infection [20]. Suicin 90–1330 is a lantibiotic that shares a high degree of homology with nisin U, which is produced by *Streptococcus uberis*. In the present study, we identified a novel bacteriocin produced by a *S*. *suis* strain isolated from a healthy carrier pig. In a complex environment colonized by a wide range of bacterial species such as the respiratory tract, bacteriocins give the producing bacteria a unique advantage that helps to ensure their persistence. The bacteriocin produced by *S*. *suis* 3908 is highly resistant to heat, pH, and proteolytic treatments, which is of great interest if the bacteriocin or the producing strain is to be used as a prophylactic therapeutic agent. On the one hand, the bacteriocin was active against all *S*. *suis* strains tested. On the other hand, the antibacterial spectrum of suicin 3908 is relatively narrow since it was active only against one (*S*. *hyicus*) of the eight swine pathogens tested that included Gram-positive and Gram-negative bacterial species. The depolarization of the cytoplasmic membrane of the indicator strain of *S*. *suis* induced by suicin 3908 was assessed using DNA-binding SYTOX green fluorescent dye. Treating the indicator strain with suicin 3908 permeabilized the cell membrane, leading to the uptake of fluorescent dye, which is in agreement with the well-known capacity of numerous bacteriocins to induce pore formation [6, 7]. We used a broth microdilution checkerboard assay to show that when suicin 3908 is combined with penicillin G or amoxicillin, which are commonly used to treat *S*. *suis* infections, additive effects were obtained. In a previous study [10], nisin A was reported to act in synergy with penicillin G.

Amino acid sequencing of the purified bacteriocin by Edman degradation revealed the presence of modified amino acid residues, suggesting that it belongs to the lantibiotic class. Suicin 3908 exhibited approximately 50% identity with three previously reported bacteriocins: bovicin HJ50 (*S*. *bovis*), thermophilin 1277 (*S*. *thermophilus*), and macedovicin (*S*. *macedonicus*). We used a PCR procedure based on the suicin 3908 N-terminus amino acid sequence and the published genome sequence of *S*. *suis* D12 (serotype 9) to amplify and sequence the complete gene locus of suicin 3908. More specifically, the cluster contained a structural gene (*suiA*), a gene (*suiM*) involved in post-translational modifications of the suicin prepeptide, a gene (*suiT*) that codes for a transporter, four immunity genes (*suiF*, *suiE*, *suiG*, and *suiI*), and two regulatory genes (*suiR* and *suiK*). *suiA* encodes a mature 33-amino-acid peptide with serine/threonine/cysteine residues that are substrates for the generation of modified amino acids and lanthionine and methyllanthionine structures. The deduced molecular mass of the unmodified peptide corresponding to suicin 3908 was 3,381 daltons, which was confirmed by the SDS-PAGE results showing that suicin 3908 migrated alongside commercial nisin A (molecular mass of 3,354 daltons). The suicin 3908 locus contained all the orthologous genes present in the bovicin HJ50, thermophilin 1277, and macedovicin loci sharing also synteny. Given that the suicin 3908 gene cluster contained a gene coding for a bifunctional enzyme (SuiM) with dehydratase and cyclase activities, suicin 3908 would be a type AII lantibiotic, which are N-terminal linear and C-terminal globular cationic peptides [21]. Wang et al. [22] recently identified a putative lantibiotic locus in the genomes of three highly virulent *S*. *suis* serotype 2 strains that caused toxic shock-like syndrome associated with two large-scale outbreaks of *S*. *suis* infections in China [2]; however, no bacteriocin activity was detected. Analysis of the sequence revealed that the putative lantibiotic modification gene *suiM* was interrupted by the insertion of a 7.9-kb integron while other biosynthesis-related genes contained various frame-shift mutations confirming the cause of the absence of bacteriocinogenic activity. A comparative analysis of the bacteriocin-encoding gene from this locus with *suiA* coding for suicin 3908 showed that they shared 100% identity suggesting that the mutational erosion of the locus described by Wang et al. [22] occurred after divergence of the common ancestor strain of 3908.

Since bacteriocin production is considered a probiotic trait, *S*. *suis* 3908 may be a candidate for use in probiotic treatment. However, in order to be used, the safety of the strain will have to be confirmed. Since this strain was isolated from a healthy carrier pig, it is likely to be a non-pathogenic isolate of *S*. *suis*. This is supported by the fact that a previous study reported that strain 3908 displays low virulence in a newborn germfree pig model and does not produce suilysin, a major virulence factor in pathogenic isolates [23]. Moreover, we showed that this strain is highly susceptible to antibiotics (penicillin G, amoxicillin) currently used in the swine industry. Taken together, these characteristics suggest that *S*. *suis* 3908 may be safe, although additional animal studies will have to be carried out to confirm this.

An advantage of bacteriocins is that they are not cytotoxic. For example, the administration of nisin A at a dietary level of 5% caused no negative effects in a rat model [24]. However, despite the fact that bacteriocins possess antibacterial properties *in vivo* and are not cytotoxic in mammals, clinical studies are sparse. While the use of bacteriocins for swine infections has not been investigated, Cao et al. [25] provided evidence for the effectiveness of nisin for treating bovine mastitis caused by *S*. *aureus*. It has also been reported that a bacteriocin produced by a strain of *Enterococcus faecium* (isolated from broiler chicken ceca) significantly reduced *Campylobacter jejuni* colonization in the chicken intestine when added to drinking water [26]. Lastly, Jabés et al. [27] documented the efficacy of an intravenous administration of the lantibiotic NAI-107 in an endocarditis rat model infected by methicillin-resistant *S*. *aureus*.

In summary, suicin 3908 is a novel narrow spectrum lantibiotic produced by a strain of *S*. *suis* serotype 2 isolated from a healthy carrier pig. The use of the purified bacteriocin or the bacteriocin producing strain may be a valuable strategy to control *S*. *suis* infections and for reducing antibiotic use by the swine industry and consequently the spread of antibiotic resistance. *In vivo* experiments confirming the ability of the strain 3908 to colonize the respiratory tract of pigs, to produce suicin 3908, and to protect against *S*. *suis* infections are currently under investigation.
